# Pyrazinamide clearance is impaired among HIV/tuberculosis patients with high levels of systemic immune activation

**DOI:** 10.1371/journal.pone.0187624

**Published:** 2017-11-02

**Authors:** Christopher Vinnard, Shruthi Ravimohan, Neo Tamuhla, Jotam Pasipanodya, Shashikant Srivastava, Chawangwa Modongo, Nicola M. Zetola, Drew Weissman, Tawanda Gumbo, Gregory P. Bisson

**Affiliations:** 1 Public Health Research Institute, New Jersey Medical School, Newark, New Jersey, United States of America; 2 University of Pennsylvania, Perelman School of Medicine, Philadelphia, Pennsylvania, United States of America; 3 Botswana-UPenn Partnership, Gaborone, Botswana; 4 Center for Infectious Diseases Research and Experimental Therapeutics, Baylor Research Institute, Baylor University Medical Center, Dallas, Texas, United States of America; Central University of Tamil Nadu, INDIA

## Abstract

Pyrazinamide is the main driver of sterilizing effect in the standard regimen in adults and older children, and this effect is concentration-dependent. Tuberculosis patients co-infected with human immunodeficiency virus (HIV) have an increased risk for poor tuberculosis treatment outcomes and adverse drug events. We sought to determine whether measures of systemic immune activation were related to pyrazinamide pharmacokinetics among HIV/tuberculosis patients. We conducted a prospective cohort study of pyrazinamide pharmacokinetics in HIV/tuberculosis patients in Gaborone, Botswana. Patients underwent intensive pharmacokinetic sampling before and after the initiation of antiretroviral therapy, which can increase immune activation in HIV/tuberculosis. Compartmental pharmacokinetic modeling was performed to determine whether variability in systemic immune activation was related to variability in pyrazinamide pharmacokinetic parameters. Forty HIV/tuberculosis patients completed the first pharmacokinetic sampling visit, and 24 patients returned for a second visit following antiretroviral therapy initiation. The pyrazinamide plasma concentration-versus-time data were best explained by a one-compartment model with first-order elimination, and a combined additive and proportional residual error model. Pyrazinamide clearance was higher in men than women. Expression of CD38 and HLA- DR on CD8+T cells, a measure of HIV-associated immune activation, was inversely related to pyrazinamide clearance, with increasing immune activation associated with decreasing pyrazinamide clearance. Future studies should verify this finding in larger numbers of tuberculosis patients with and without HIV co-infection.

## Introduction

Pyrazinamide is the key sterilizing-effect drug in the first-line tuberculosis treatment regimen [1–4]. Four prospective clinical studies have demonstrated that pyrazinamide efficacy is concentration driven, with the sterilizing effect and long-term outcomes driven by peak concentrations [1,2], the 24-hour area under the concentration-time curve (AUC_0-24_) [3], or the AUC_0-24_ to minimum inhibitory concentration ratio [4]. These findings are also consistent with preclinical studies [5]. Given that the AUC is inversely proportional to drug clearance, and the peak concentration to the volume of distribution, the main drivers of clinical outcome will be the variability in these pharmacokinetic parameters.

Tuberculosis patients co-infected with HIV have an increased risk for poor tuberculosis treatment outcomes, which include delays in achieving sputum sterilization, episodes of relapse after completing tuberculosis therapy, development of acquired drug resistant tuberculosis during therapy, and death [6–9]. Furthermore HIV-infected patients have an increased risk for adverse drug events during tuberculosis therapy. As an example, hepatotoxicity is a common occurrence during the treatment of active tuberculosis patients with regimens that include pyrazinamide, and HIV co-infection is an independent risk factor for this adverse outcome [10]. Among patients with HIV/tuberculosis co-infection, more advanced HIV disease (as defined by World Health Organization staging) is an additional predictor of hepatotoxicity [11]. The potential for treatment-related hepatotoxicity places a burden on tuberculosis control programs, and contributes to treatment interruptions [12].

HIV-associated tuberculosis is characterized by cellular immune activation and high levels of circulating pro-inflammatory cytokines [13]. As a measure of cellular immune activation, the percent of CD8+ T cells co-expressing CD38 and HLA-DR (%CD38^+^DR^+^CD8^+^) predicts HIV disease progression better than CD4^+^ T cell counts or HIV viral loads [14,15]. High levels of systemic immune activation during chronic HIV infection is accompanied by markers of bacterial translocation, including soluble CD14 (sCD14), a marker of monocyte activation in response to bacterial lipopolysaccharide (LPS) [16]. Most HIV/tuberculosis patients have reductions in immune activation during treatment for both infections. However, some patients, particularly those with acquired immunodeficiency syndrome (AIDS) who start antiretroviral therapy very soon after tuberculosis diagnosis, can have rapid increases in immune activation on treatment, resulting in overt signs of inflammation [13,17].

We have previously demonstrated that increasing levels of HIV-associated immune activation were associated with impaired isoniazid clearance in a cohort of HIV/tuberculosis patients in Botswana, with increased isoniazid serum concentrations among patients with greater immune activation [18]. Immune activation and pro-inflammatory cytokines are also known to regulate the expression and activity of some phase I xenobiotic metabolic enzymes and drug transporters, but effects on pyrazinamide pharmacokinetics are unknown [19,20]. Patients with high levels of inflammation and immune activation due to other causes, such as bacterial sepsis or acute viral infections, demonstrate impaired drug metabolizing capacity and increased pharmacokinetic variability [21,22]. We sought to determine whether levels of cellular and systemic immune activation would be associated with impaired pyrazinamide clearance among HIV/tuberculosis patients.

## Methods

### Study design

We conducted a prospective study of pyrazinamide pharmacokinetics among HIV/tuberculosis patients in Gaborone, Botswana.

### Study population

HIV-infected adults (21 years of age and older, following Botswana Ministry of Health criteria for informed consent) were eligible for enrollment if they were citizens of Botswana, naïve to antiretroviral therapy, and newly diagnosed with pulmonary tuberculosis. Patients must have been initiated on a standard first-line tuberculosis treatment regimen, following World Health Organization guidelines for weight-based dosing bands, administered under directly observed therapy [23]. The diagnosis of pulmonary tuberculosis must have been established by either a positive sputum smear, a positive GeneXpert MTB/RIF assay (Cepheid, Sunnyvale, CA, USA), or the presence of World Health Organization criteria for smear-negative pulmonary tuberculosis. Exclusion criteria included pregnancy, renal insufficiency (defined as a creatinine clearance less than 50 mL/min), and hepatic dysfunction (defined as either an alanine transaminase or aspartate transaminase greater than 3 times the upper limit of normal). The study cohort has been previously described in a report of isoniazid population pharmacokinetics [18].

### Data collection

The study procedures consisted of two pharmacokinetic study visits, with each visit conducted at the Infectious Disease Care Clinic at Princess Marina Hospital, located in the capital city of Gaborone. The first pharmacokinetic study visit occurred between 5 and 28 days after the initiation of anti-tuberculosis therapy, corresponding to steady-state conditions during the intensive phase of tuberculosis treatment. All participants were eligible to return for a second pharmacokinetic visit during the intermittent phase of tuberculosis treatment, provided that antiretroviral therapy had also been initiated. All patients initiated antiretroviral therapy with the combination of tenofovir/emtricitabine/efavirenz. The procedures for each pharmacokinetic study visit were identical. After an overnight fast, oral doses of the anti-tuberculosis drugs were obtained from the Gaborone City Clinic and directly administered to the participant under directly observed therapy on the morning of the pharmacokinetic visit. Pharmacokinetic blood samples (10 mL) were drawn pre-dose, and then 0.3, 0.9, 2.2, 4.5, and 8 hours post-dosing. Covariate information, obtained either from review of the clinic chart or direct measurement, included age, weight, body mass index (BMI), gender, serum creatinine, HIV viral load, and CD4^+^ T cell count. HIV-associated systemic immune activation was measured as %CD38^+^DR^+^CD8^+^, and with plasma levels of Interleukin 6 (IL-6) and C-reactive protein (CRP). We also measured sCD14 as a surrogate for microbial translocation in the plasma.

### Analytical methods

Blood samples were transported to the Botswana Harvard Partnership Laboratory upon collection for plasma and peripheral blood mononuclear cell isolation using Ficoll-Paque Plus (GE Healthcare) density gradient centrifugation. Plasma, previously frozen at -80°C, was diluted 500-fold to determine concentrations of sCD14 (R&D Systems, Minneapolis, MN, USA). Undiluted plasma was used to determine concentrations of IL-6 (R&D Systems). C-reactive protein levels were determined after diluting plasma between 1:1000 and 1:50,000 (R&D Systems). Assays were conducted as per manufacturers’ protocol. We determined %CD38^+^DR^+^CD8^+^ T cells using previously described methods [18].

We used a stable-isotope dilution liquid chromatography-electrospray ionization tandem mass spectrometry (LC-ESI-MS/S) method to determine pyrazinamide concentrations in serum samples. Pyrazinamide and the stable isotope labeled pyrazinamide-d3 were purchased from Sigma (St. Louis, MO, USA) and CDN Isotopes (Quebec, Canada), respectively. A 6-point calibration curve was prepared and each analytical run included calibrators, controls, and internal standards for quantitation of pyrazinamide in the patient samples. The lower limit of quantification of this method was 0.6 mg/L with a correlation coefficient 0.99 (4.1% intra-day precision). Briefly, 10 μL serum samples were added to 190 μL 0.2% formic acid in water. The samples were further 10-fold diluted using 0.2% formic acid in water containing 4 μg/mL labeled-isotope internal standards. Chromatographic separation was attained on an Acquity UPLC HSS T3 1.8 μm 50 x 2.1 mm analytical column (Waters) maintained at 30°C at a flow of 0.2 mL/min with a binary gradient. The solvents for UPLC and gradient condition were as described in our previous publication [18]. Pyrazinamide was detected by tandem mass spectrometry (MS/MS) using positive electrospray ionization (ESI), complete LC, Turbulon source, mass transitions, and optimized collision-induced dissociation conditions. Shimadzu Nexera Ultra High Performance Liquid Chromatography interfaced with a Sciex 5500QTRAP mass spectrometer was used for sample injection and chromatographic separation. Analyst 6.1 software was used for data analysis.

### Statistical analysis

Nonlinear mixed effects modeling was performed to estimate the pharmacokinetics parameters [24]. Model development used first-order conditional estimation (FOCE) in Phoenix NLME 7.0 (Certara) to provide estimates of the typical values for population pharmacokinetic parameters, between-subject variability, inter-occasional variability, and residual variability.

Serum concentration-versus-time data were fitted to 1- and 2-compartment models with first-order elimination. Between-subject variability was evaluated with an exponential variability model with mean of zero and variance ω^2^. Inclusion of between-subject variability was evaluated individually for each pharmacokinetic parameter, and inclusion in the model was based on reduction in the objective function value corresponding to p<0.05. Interoccasional variability on each parameter was modeled with a mean of zero and variance κ^2^. Additive, proportional, and combined residual error models were tested to describe residual unexplained variability. Covariance between each parameter’s between-subject variability was evaluated in a variance-covariance matrix. Pyrazinamide absorption was assessed with a first-order absorption model and a transit compartment model. Plots of the random effects on pharmacokinetic parameters versus the individual covariates provided insight into potential covariate effects. Goodness-of-fit was examined visually with plots of observed versus population predicted concentration, observed versus individual predicted concentrations, individual weighted residuals versus individual predicted concentrations, and conditional weighted residuals versus time.The final model was selected based on biologic plausibility, goodness-of-fit as evidenced by visual examination of diagnostic plots, and statistical criteria.

Visual predictive checks were used to evaluate the performance of candidate and final models for pyrazinamide pharmacokinetics, by simulation of 1,000 datasets. The distribution (median, 5^th^, and 95^th^ percentiles) of the simulated concentrations versus time were compared with the distribution of the observed values in the original dataset. Differences and overlap of the simulated and original distributions indicated the accuracy of the identified model.

## Results

### Pyrazinamide pharmacokinetic model development

**Fig 1:**
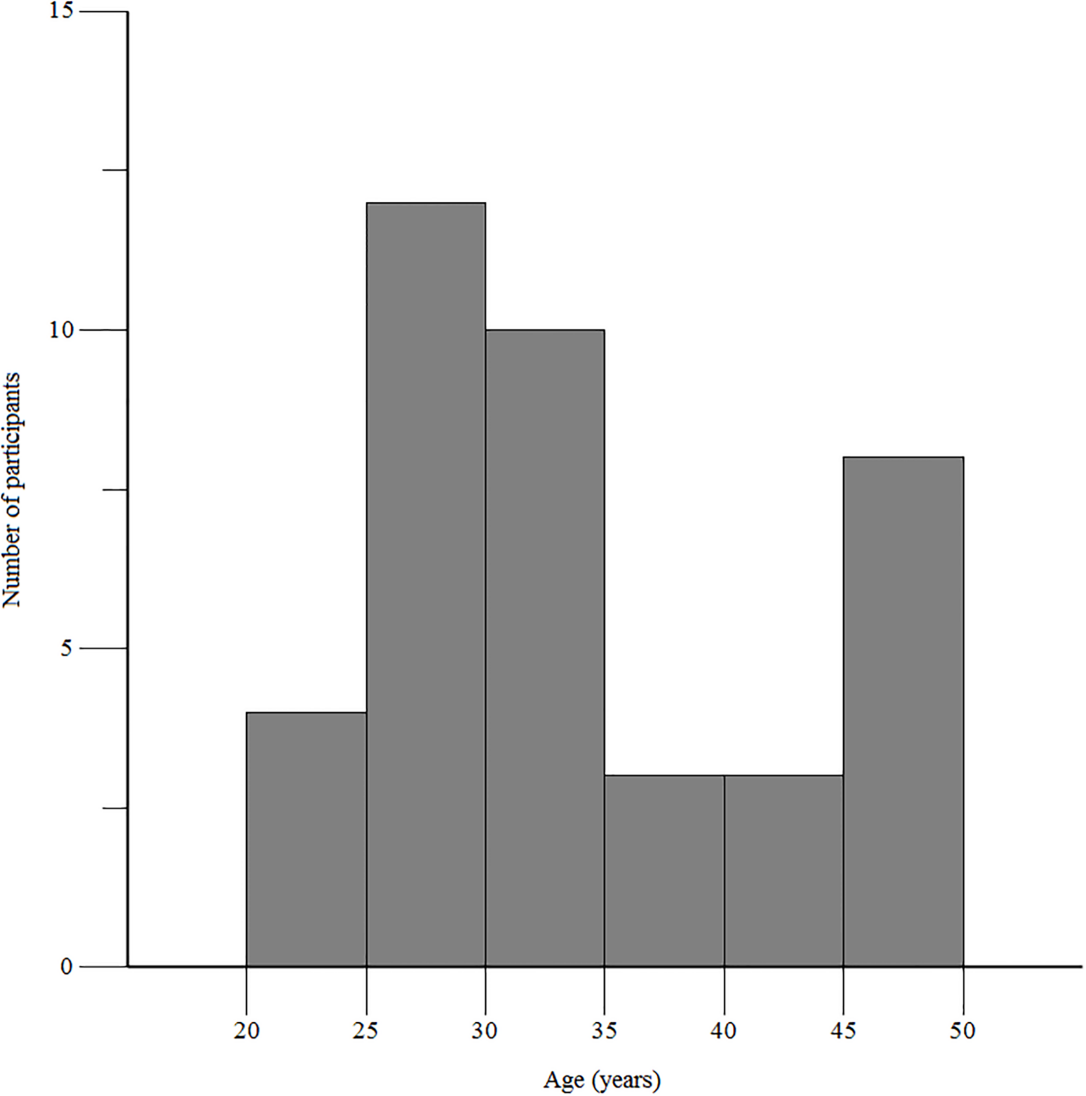
Histogram of age distribution of study participants.

Out of 61 patients screened, 40 patients were enrolled in the study and completed the first pharmacokinetic study visit. Twenty-four patients returned for the second pharmacokinetic study visit, performed approximately 4 weeks after the initiation of antiretroviral therapy, and 9 of 24 patients were receiving pyrazinamide at the time of the second pharmacokinetic study visit. Across both visits, the pyrazinamide pharmacokinetic dataset included 294 concentration-time points for analysis. The median age of the study population was 32 years (Fig 1), with 55% male, and the median CD4^+^ T cell count was 238 cells per uL (interquartile range 105 to 339). Clinical and demographic characteristics of study participants have been previously reported [18].

**Fig 2:**
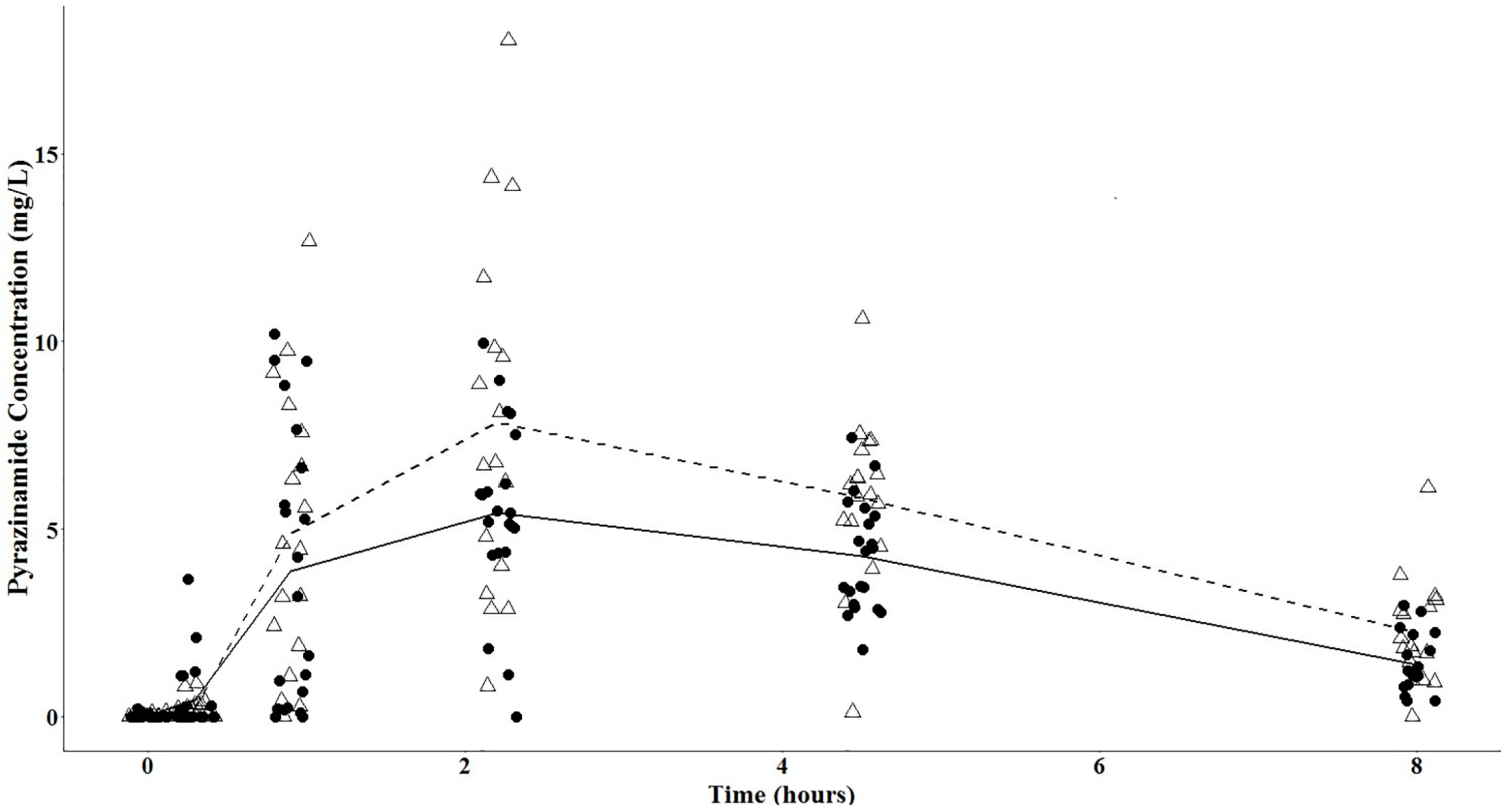
Mean pyrazinamide concentration-versus-time curves prior to the initiation of antiretroviral therapy. Legend: open triangles: women, closed circles: men; dashed line: mean pyrazinamide concentrations among women; solid line: mean pyrazinamide concentrations among men.

**Fig 3:**
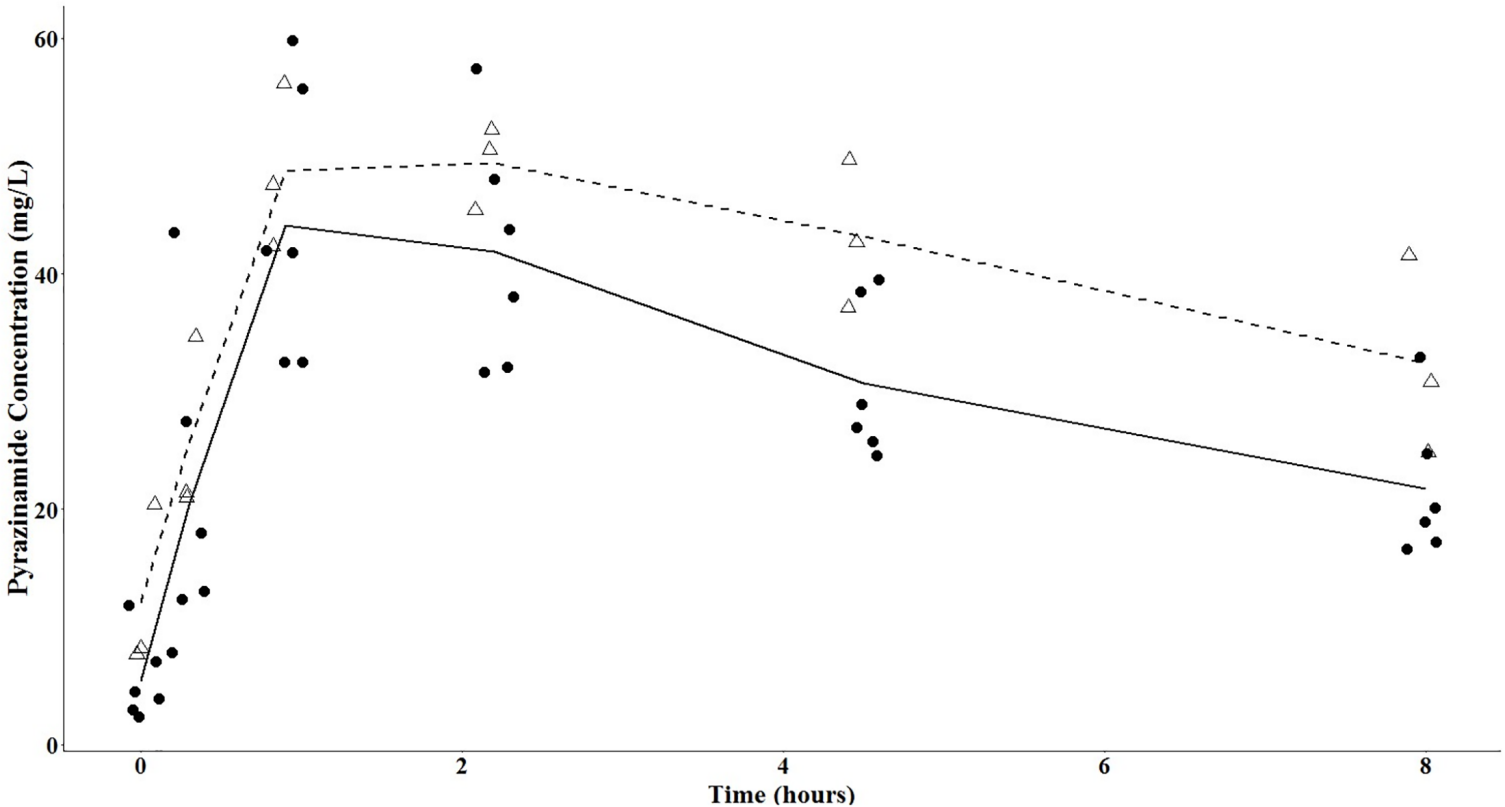
Mean pyrazinamide concentration-versus-time curves following the initiation of antiretroviral therapy. Legend: open triangles: women, closed circles: men; dashed line: mean pyrazinamide concentrations among women; solid line: mean pyrazinamide concentrations among men.

**Fig 4:**
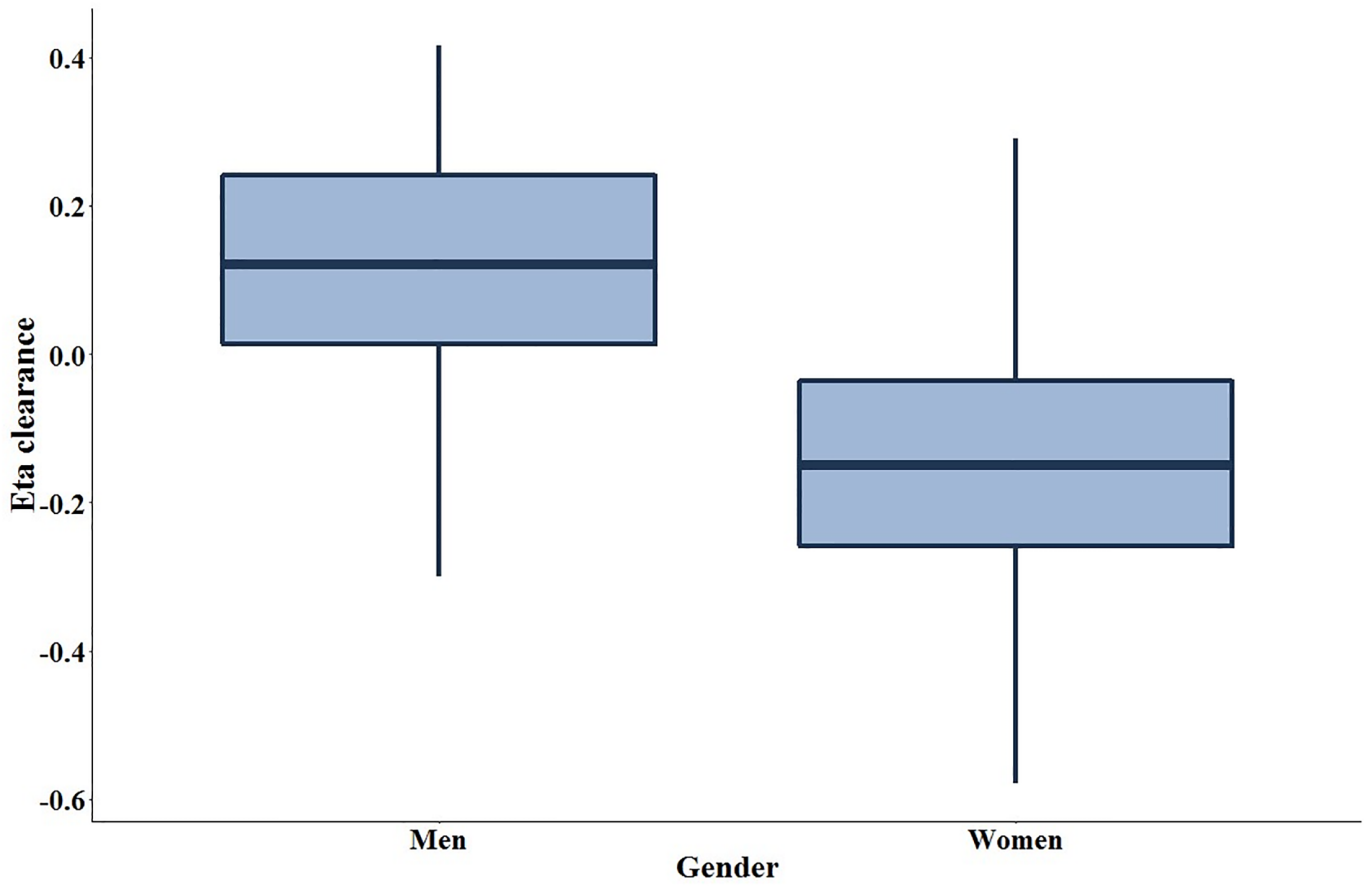
Eta covariate box plot of gender versus between-subject variability in pyrazinamide clearance (ηCL). The dots represent individual values.

The pyrazinamide mean concentration-versus-time plots are shown in Figs 2 and 3, with separate plots for men and women, before and after initiation of antiretroviral therapy. The pyrazinamide serum concentration-versus-time data were best fit with a 1-compartment model with first-order elimination, and a combined additive and proportional residual error model. The structural model supported between-subject variability on the volume of distribution, clearance, and the absorption coefficient, and interoccasional variability on the volume of distribution. The eta-covariate plot for gender and clearance supported a covariate relationship (Fig 4). The addition of gender into the pharmacokinetic model as a covariate on clearance led to a reduction of 11.89 in the objective function value (p-value <0.001 under a chi-squared distribution with 1 degree-of-freedom), and a reduction in between-subject variability in clearance from 27.4% to 22.4%. We introduced allometric scaling of body weight (measured at each visit) on clearance and volume, which further reduced the objective function value by 9.68 (p-value 0.008 under a chi-squared distribution with 2 degrees-of-freedom). We did not observe significant covariate effects with age or baseline creatinine clearance.

### Covariate effects of systemic immune activation parameters

**Fig 5:**
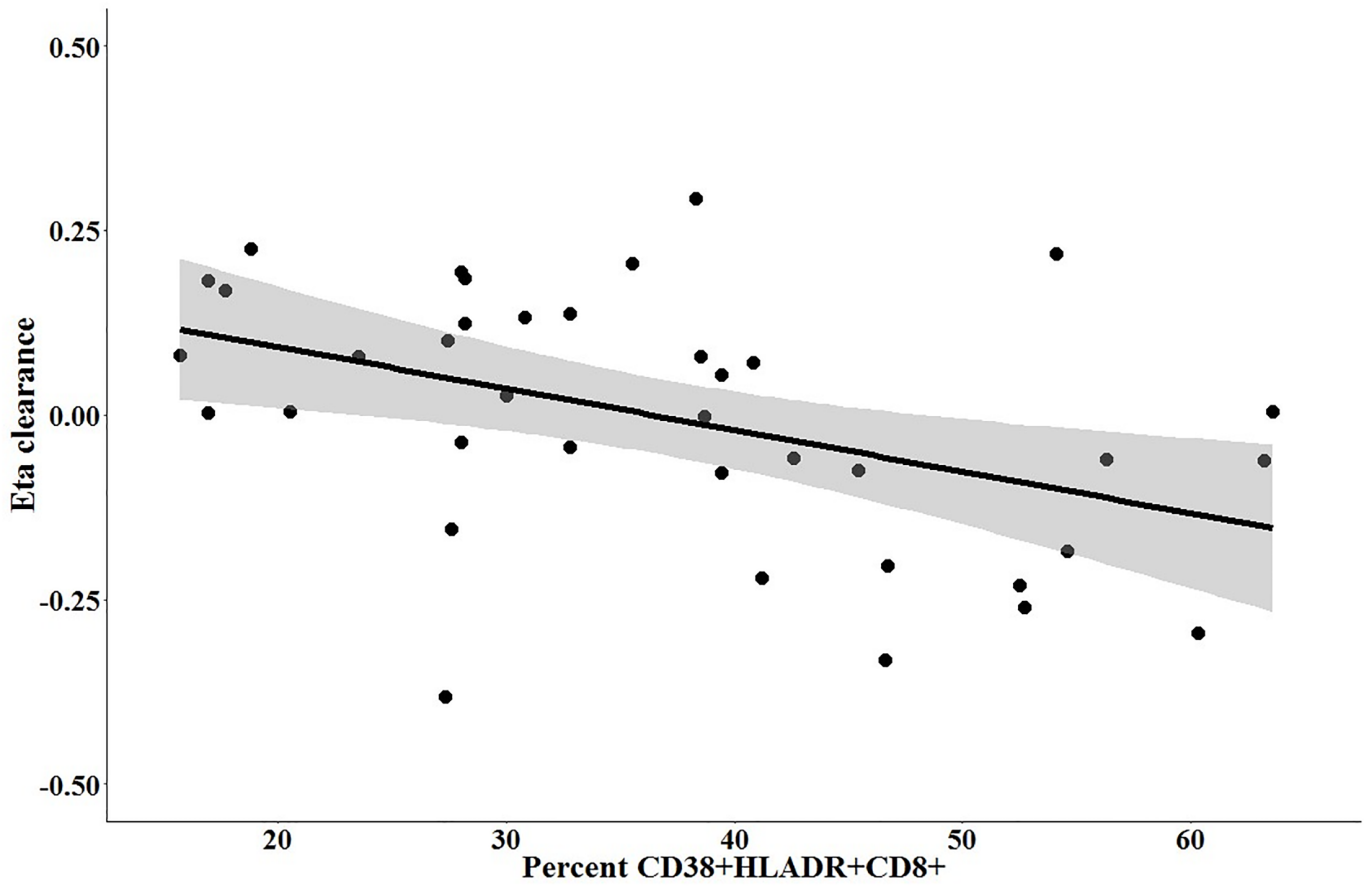
Eta covariate plot of %CD38^+^HLADR^+^CD8^+^ versus between-subject variability in pyrazinamide clearance (ηCL). The dots represent individual values.

Next, we evaluated markers of HIV disease as covariates in the pharmacokinetic model. We did not observe covariate effects for CD4+ T cell counts, HIV viral load, sCD14, IL-6, CRP, or sCD14 on the pharmacokinetic parameters for volume of distribution or clearance. The eta-covariate plot relating %CD38^+^DR^+^CD8^+^ and pyrazinamide clearance is shown in Fig 5. The negative slope of the regression line indicated that increasing levels of %CD38^+^DR^+^CD8^+^ were associated with decreasing pyrazinamide clearance, independent of sex and weight. As shown in Table 1, the inclusion of %CD38^+^DR^+^CD8^+^ as a covariate on clearance led to a 5.91 reduction in the objective function value (p = 0.015 under a chi-squared distribution with 1 degree of freedom), and the between-subject variability in clearance was further reduced from 21.3% to 17.5%.

**Table 1:** Evaluation of markers of systemic immune activation as covariates on pyrazinamide clearance.

HIV disease marker evaluated as covariate on pyrazinamide clearance	Change in objective function value	Between subject variability of clearance
Base model*	-	21.3%
Percent CD38^+^HLADR^+^CD8^+^	5.91 **	17.5%
Log-IL6	0.09	21.2%
sCD14	1.22	21.1%
CD4+ T cell count	0.01	21.5%
Log HIV viral load	0.37	20.9%

*Includes the covariate effects of gender and weight on clearance.

**p = 0.015 under a chi-squared distribution with 1 degree of freedom

**Fig 6:**
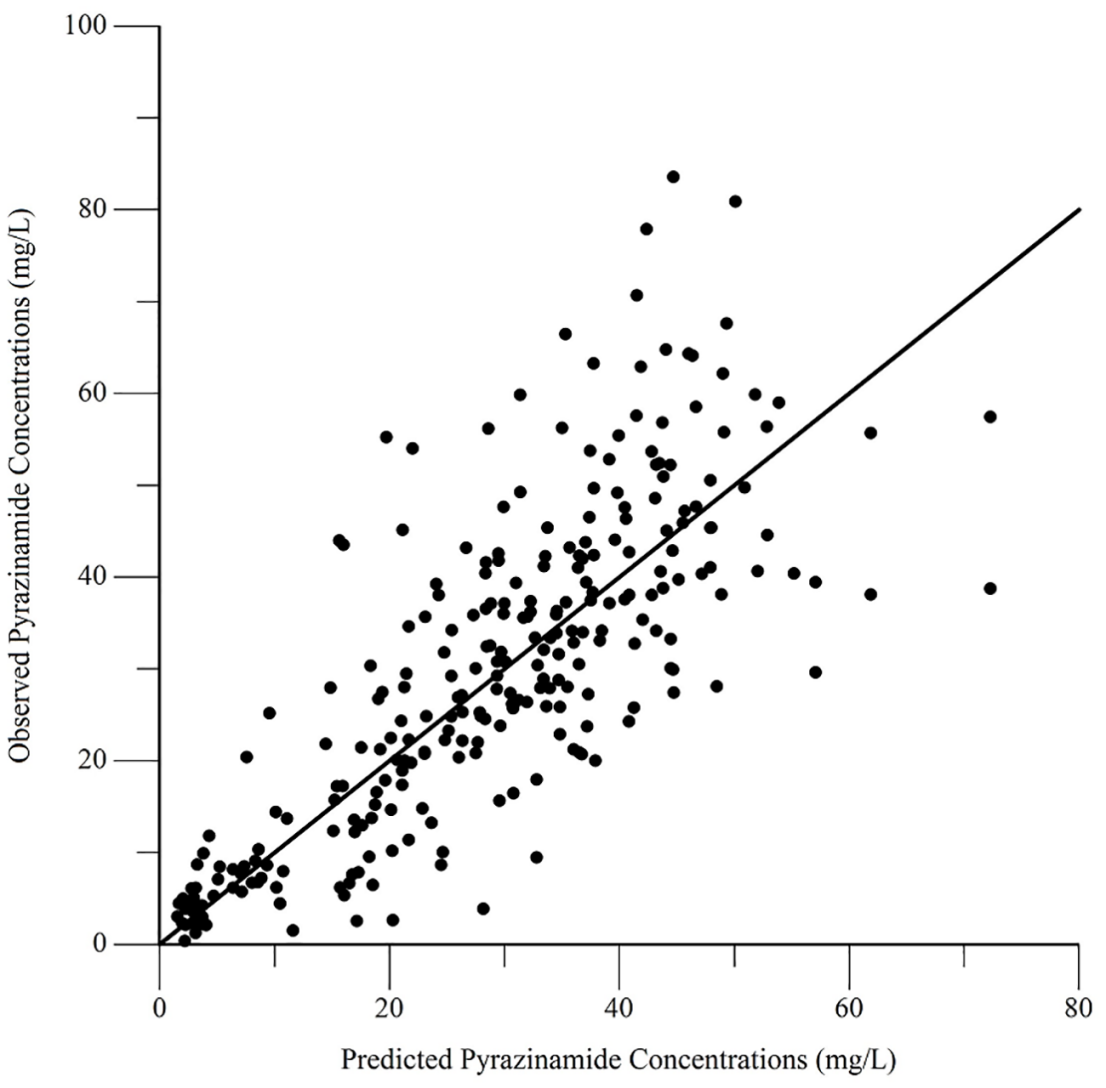
Diagnostic plot of observed versus population predicted pyrazinamide concentrations.

**Fig 7:**
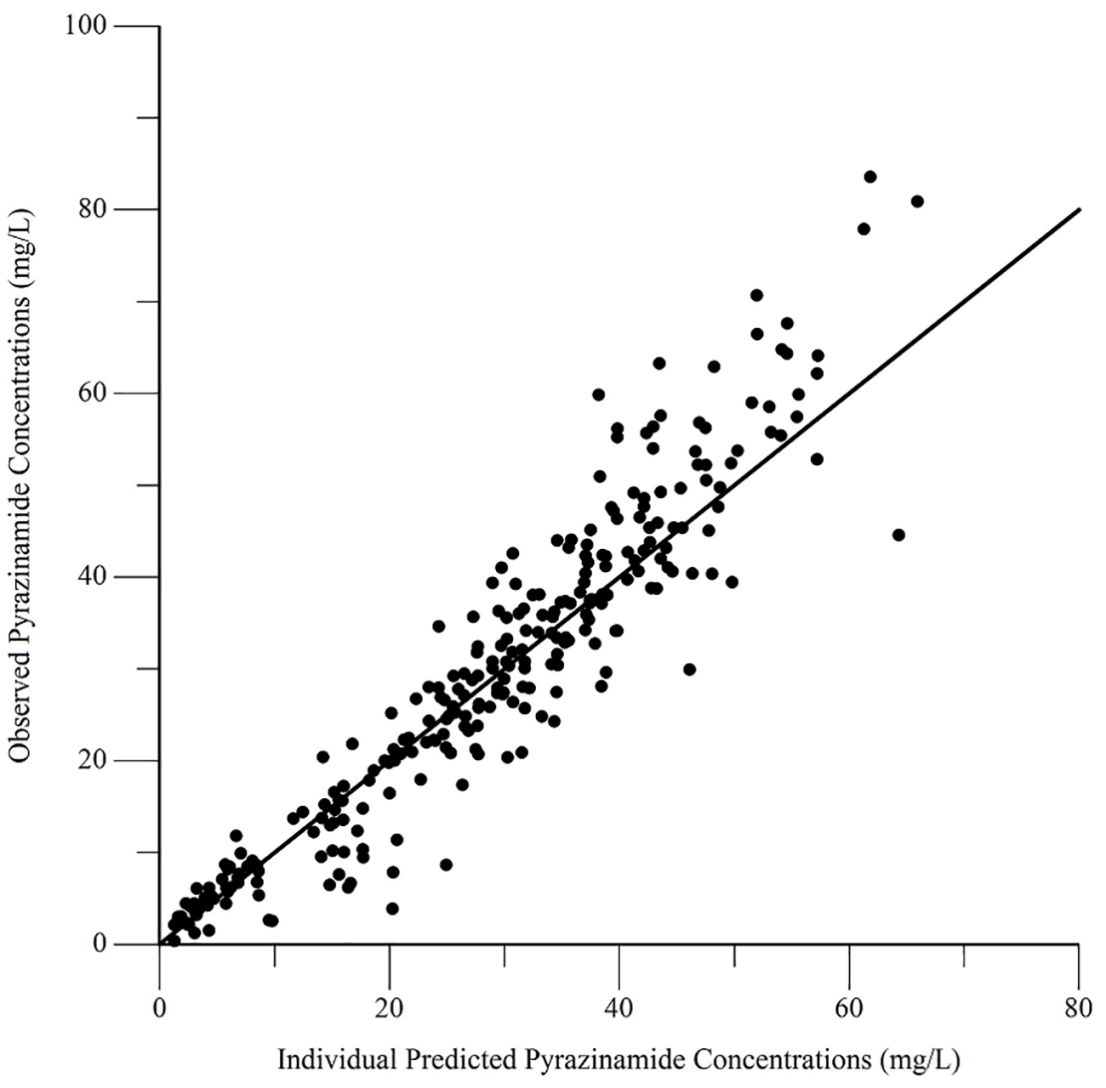
Diagnostic plot of observed versus individual predicted pyrazinamide concentrations.

**Fig 8:**
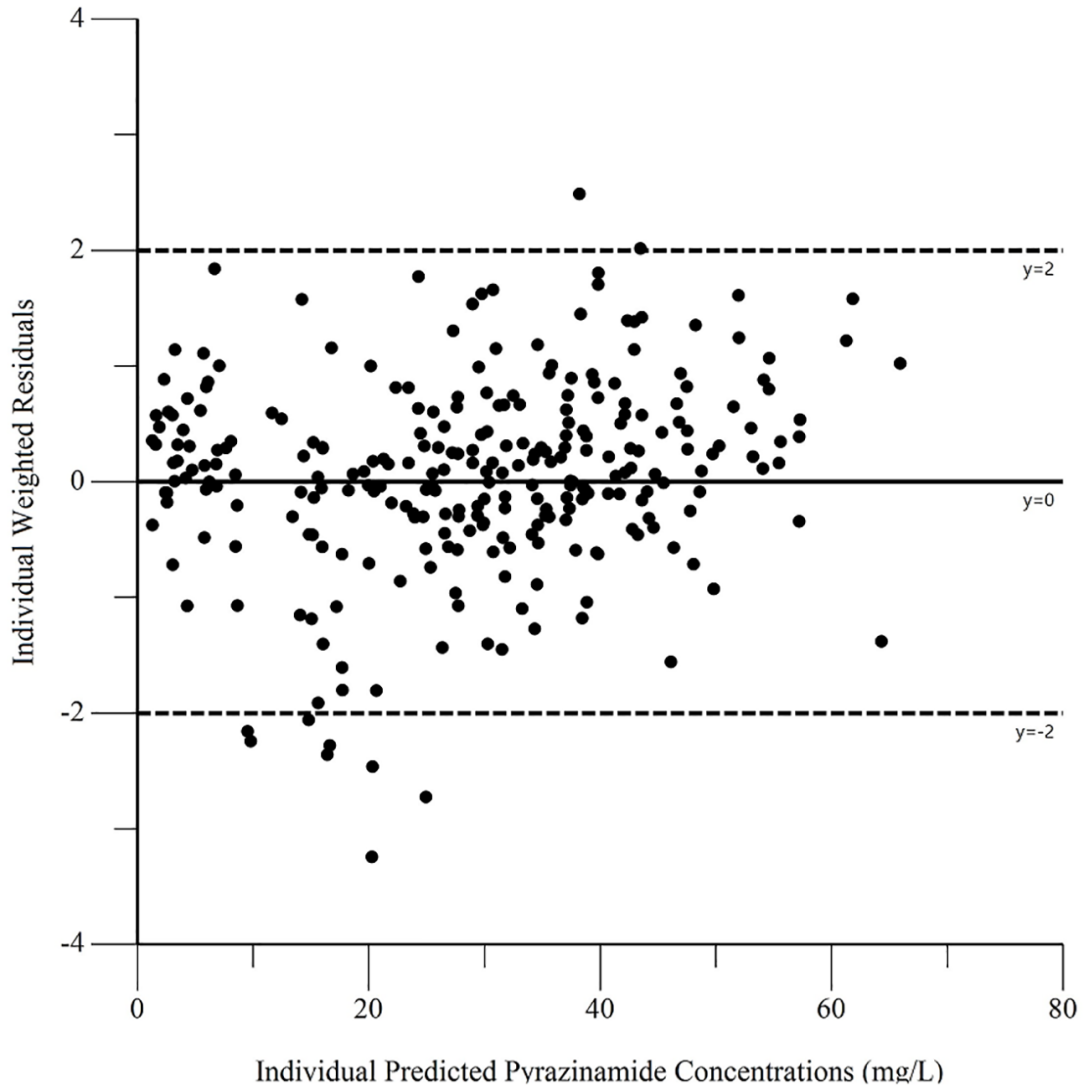
Diagnostic plot of individual weighted residuals versus individual predicted pyrazinamide concentrations.

**Fig 9:**
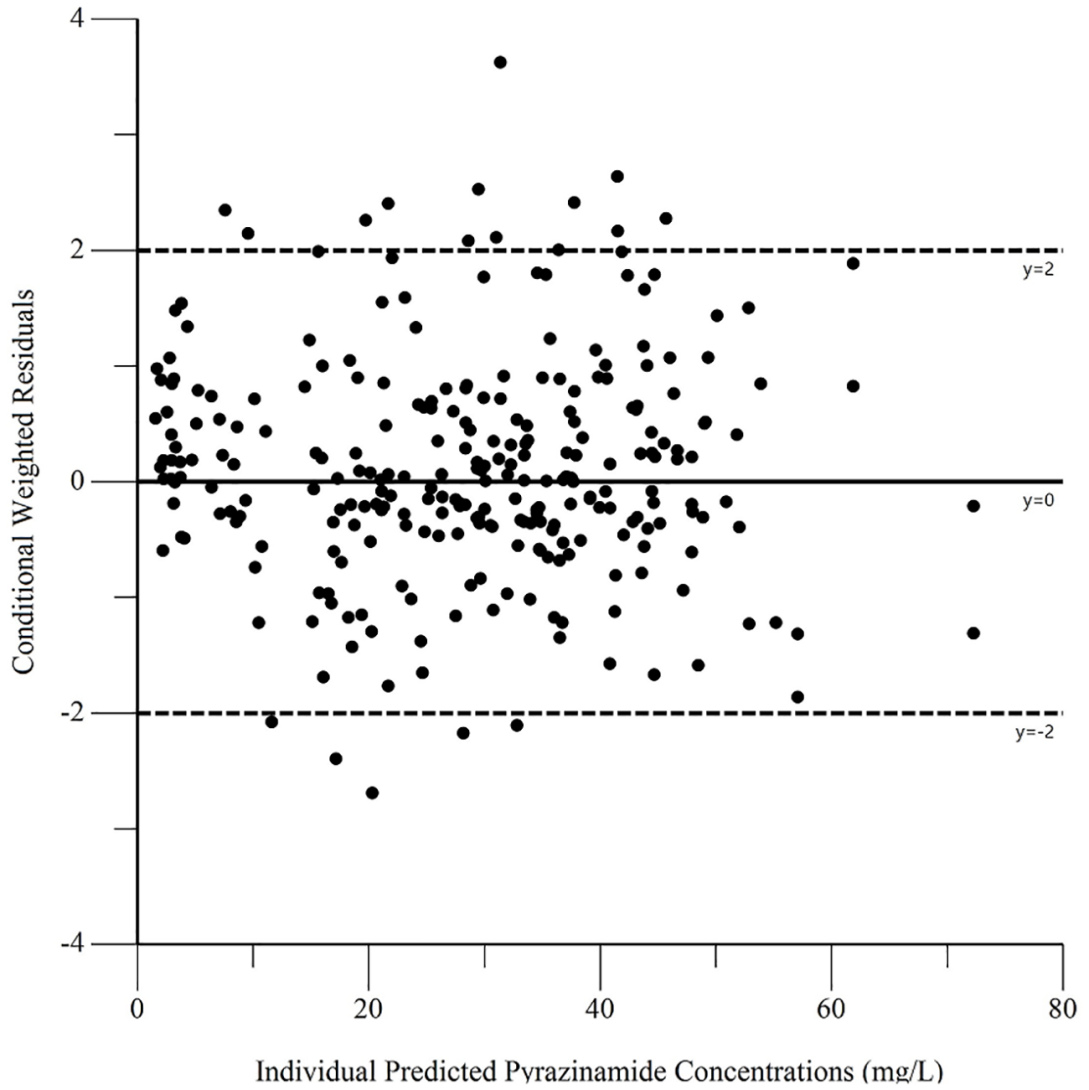
Diagnostic plot of conditional weighted residuals versus predicted concentrations.

**Fig 10:**
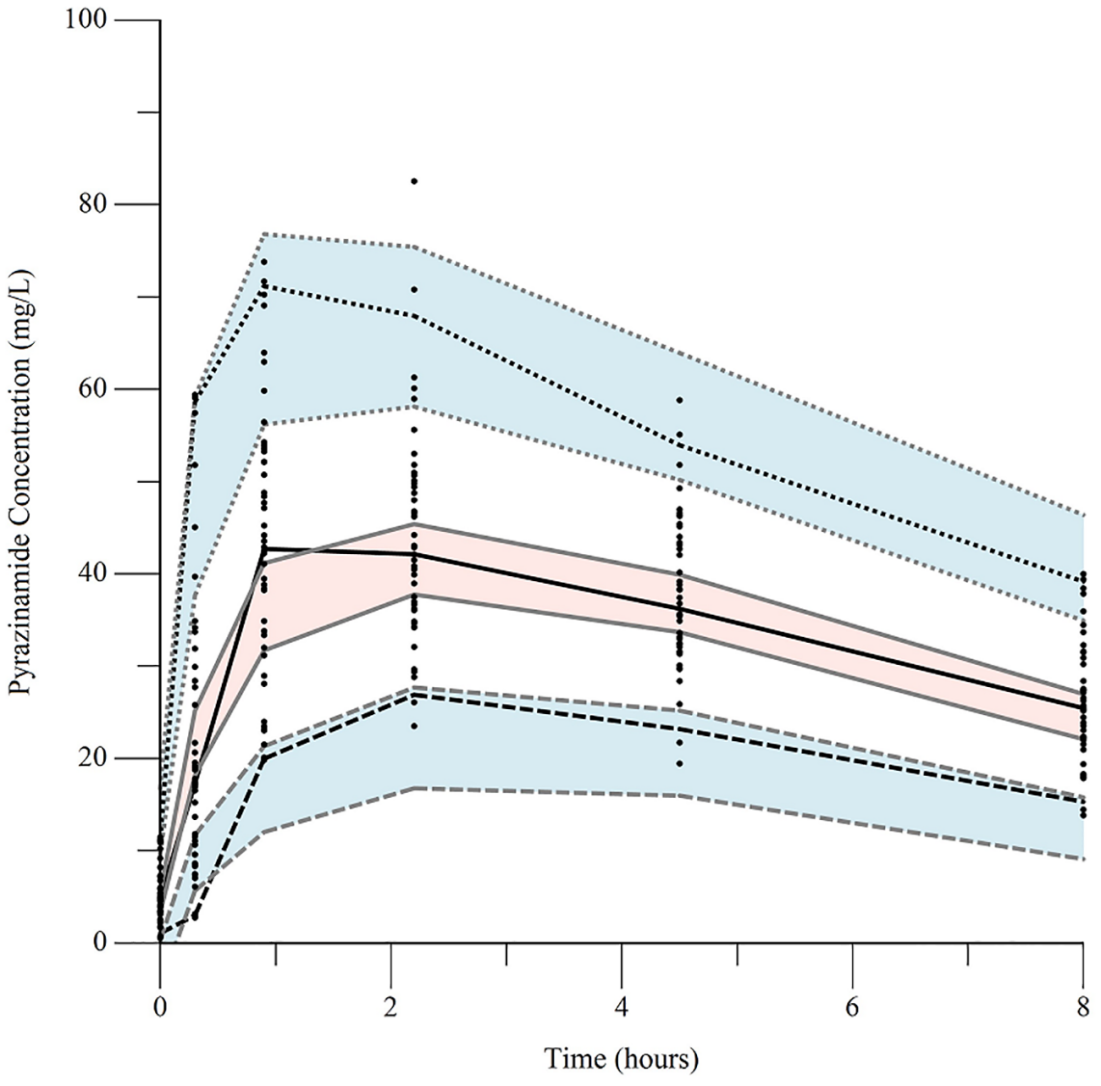
Visual predictive check of final pharmacokinetic model with 1000 replicates. Dotted line: observed 5^th^ percentile; solid line: observed 50^th^ percentile; dashed line: observed 95^th^ percentiles; blue shaded region: 95% confidence intervals for predicted 5^th^ and 95^th^ percentiles; pink shaded region: 95% confidence interval for 50^th^ percentile.

The final pharmacokinetic parameters are shown in Table 2. Epsilon shrinkage for the final model was 15.2%, eta shrinkage was 20.4% for clearance, 40.5% for the volume of distribution, and 14.4% for the absorption rate constant. Diagnostic plots for the model are shown in Figs 6–9, and the visual predictive check is shown in Fig 10.

**Table 2:** Final model parameter estimates for the population pharmacokinetics of pyrazinamide in adult HIV/tuberculosis patients.

Population PK Parameters	Typical Value (% RSE)	Median (95% CI)
Absorption rate constant (Ka, hr^-1^)	1.31 (14.14)	1.32 (1.06, 1.70)
Apparent oral clearance (CL/F, L/hr)	3.52 (3.83)	3.49 (3.23, 3.73)
Apparent volume of distribution (V/F, L)	28.57 (9.07)	28.35 (25.56, 32.13)
Covariate effect of weight on apparent oral clearance (Θ_CL/F,WT_)	0.75*	0.75*
Covariate effect of weight on apparent volume of distribution (Θ_V/F,WT_)	1*	1*
Covariate effect of gender on apparent oral clearance (Θ_CL/F,gender_)	-0.40 (20.44)	-0.40 (-0.51, -0.23)
Covariate effect of cellular immune activation on apparent oral clearance (Θ_CL/F, %CD38+HLADR+CD8+_)	-0.22 (30.38)	-0.24 (-0.40, -0.07)
Between-Subject Variability		
Between-subject variability of the absorption rate constant (%)	68.5% (22.7)	67.5% (43.5, 90.1)
Between-subject variability of clearance (%)	17.5% (6.3)	16.6% (9.8, 22.4)
Between-subject variability of the volume of distribution (%)	18.2% (10.1)	16.8% (4.1, 27.9)
Inter-Occasional Variability		
Inter-occasional variability of the volume of distribution (%)	11.7% (10.2)	10.8% (1.6, 19.0)
Residual error		
Proportional error (% CV)	0.22 (33.94)	0.21 (0.12, 0.28)
Additive error (SD, ug/mL)	2.41 (96.36)	2.43 (0.72, 3.97)

CL = clearance; V = Volume of distribution of central compartment; Ka = Absorption rate constant

*Fixed

## Discussion

Previous studies of pyrazinamide pharmacokinetics in HIV/tuberculosis patients have had limited success in identifying HIV disease markers predictive of pyrazinamide concentrations [2,25,26]. By reframing the HIV disease variables in terms of systemic immune activation, which predicts HIV disease progression better than CD4^+^ T cell counts or HIV viral loads [15–17], we provide a novel paradigm for understanding the sources of pharmacokinetic variability among HIV/tuberculosis patients. Among HIV/tuberculosis patients in Botswana who were ART-naïve at the time of enrollment, we observed that increasing levels of HIV-associated cellular immune activation, as defined by %CD38^+^DR^+^CD8^+^, were associated with decreasing pyrazinamide clearance. In addition, we observed that pyrazinamide clearance was increased among men, leading to lower systemic pyrazinamide exposures among men compared with women.

Pyrazinamide is a pro-drug that is metabolized by *M*. *tuberculosis* to pyrazinoic acid, which exerts a potent sterilizing effect on tuberculosis lesions through uncertain mechanisms [27]. Recently, it was demonstrated that host enzymes also convert pyrazinamide to pyrazinoic acid [28]. In support of a role for hepatic amidase activity in pyrazinamide metabolism, tuberculosis patients with hepatic impairment demonstrate a profound reduction in pyrazinamide clearance [29]. Both pyrazinamide and pyrazinoic acid are metabolized by xanthine oxidase to form the 5-hydroxy metabolites, and xanthine oxidase activity does not appear to be the rate-limiting step in this pathway [30]. Thus, systemic pyrazinamide clearance may be impaired by processes that negatively regulate hepatic amidase activity.

Recently, we have demonstrated that increasing %CD38^+^DR^+^CD8^+^ is related to reduced clearance of isoniazid, which is metabolized by N-acetyltransferase-2 (NAT2) and CYP2E1 [18]. Together with the current findings, we have identified a single measure of HIV-associated cellular immune activation that is related to impaired clearance of both isoniazid and pyrazinamide. This novel relationship can be placed in the broader context of the well-described inhibitory effects of inflammation on drug metabolism. It is noteworthy that a measure of chronic cellular immune activation related to pyrazinamide clearance, while various markers of acute inflammation (IL-6 and CRP) did not. Given that the relationship between serum concentrations and efficacy has been well-established for pyrazinamide in the treatment of tuberculosis, with successful outcome related both to the peak concentration [1,2] and the AUC [3,4], patient factors that contribute to variability in pharmacokinetic exposure will impact clinical outcomes. In addition, longitudinal changes in cellular immune activation during treatment will be reflected in changes in pyrazinamide clearance, which is inversely proportional to the AUC.

The observation that immune activation is inversely related to pyrazinamide clearance is also noteworthy in the context of anti-tuberculosis drug hepatotoxicity, and the increased risk that has been observed among HIV/tuberculosis patients. Interestingly, Tostmann *et al* have demonstrated that pyrazinamide itself, rather than its 5-hydroxy metabolite, is involved in hepatotoxicity [31]. Furthermore, pre-treatment with isoniazid increases the toxicity of pyrazinamide in the hepatoma cell model [32]. A covariate effect that inhibits the clearance of both isoniazid and pyrazinamide could influence hepatotoxicity risk via these synergistic effects. As an alternate explanation, the immune response may drive pyrazinamide hepatotoxicity, which would explain the failure to link pyrazinamide pharmacokinetic exposures to hepatotoxicity in a toxicodynamic analysis of controlled trials [33]. Parent-metabolite toxicodynamic modeling may provide insight into the relationship, if any, between impaired pyrazinamide metabolism and liver injury.

We observed a relationship between gender and pyrazinamide clearance, with higher clearance (and hence lower pyrazinamide AUC) among men. Prior studies of the effect of gender on pyrazinamide pharmacokinetics have demonstrated similar findings. In a study of adult tuberculosis patients in India (predominantly HIV-uninfected) using non-compartmental pharmacokinetic analysis, the pyrazinamide C_max_ and AUC_0-8_ were reduced 14% and 10%, respectively, among men compared with women [34]. Among HIV/tuberculosis patients in South Africa, the pyrazinamide AUC_0-12_ among men was reduced 14% [25] compared with women. In our study, the covariate effect of gender on pyrazinamide clearance remained significant after adjusting for differences in body weight. Additional work is needed to elucidate the mechanism underlying this relationship.

This study had several important limitations. First, the study was not powered to study the relationship of pyrazinamide pharmacokinetics with tuberculosis treatment outcomes, such as treatment success or time to sputum sterilization. In addition, the study was not designed to capture episodes of adverse pyrazinamide effects, such as hepatotoxicity. Although we postulate that the observed relationship between HIV-associated immune activation and pyrazinamide clearance is related to an inhibition of liver amidase activity, the clinical study was not designed to investigate this potential mechanism. Strengths of the study include the novel use of HIV-associated immune activation markers in a population pharmacokinetic modeling framework, underpinned by a proposed mechanistic relationship between the immune response and drug metabolism.

## Conclusions

In summary, a single measure of HIV-associated immune activation, %CD38^+^DR^+^CD8^+^, was related to pyrazinamide exposure in a cohort of HIV/tuberculosis patients in Botswana. Future studies should explore this relationship among tuberculosis patients with and without HIV co-infection, and investigate cellular mechanisms for the interaction between inflammation and drug metabolism.

## Ethics

The study was approved by institutional review boards of Ministry of Health of Botswana and the University of Pennsylvania. We obtained written informed consent from all participants.

## Supporting information

S1 Table Anonymized dataset of pyrazinamide pharmacokinetics. (CSV)
